# Capsid opening enables genome release of iflaviruses

**DOI:** 10.1126/sciadv.abd7130

**Published:** 2021-01-01

**Authors:** Karel Škubník, Lukáš Sukeník, David Buchta, Tibor Füzik, Michaela Procházková, Jana Moravcová, Lenka Šmerdová, Antonín Přidal, Robert Vácha, Pavel Plevka

**Affiliations:** 1 Central European Institute of Technology, Masaryk University, Kamenice 753/5, 625 00 Brno, Czech Republic.; 2 Department of Condensed Matter Physics and National Centre for Biomolecular Research, Faculty of Science, Masaryk University, Kamenice 753/5, 625 00 Brno, Czech Republic.; 3 Department of Zoology, Fishery, Hydrobiology, and Apidology, Faculty of Agronomy, Mendel University in Brno, Zemědělská 1/1665, 613 00 Brno, Czech Republic.

## Abstract

The family Iflaviridae includes economically important viruses of the western honeybee such as deformed wing virus, slow bee paralysis virus, and sacbrood virus. Iflaviruses have nonenveloped virions and capsids organized with icosahedral symmetry. The genome release of iflaviruses can be induced in vitro by exposure to acidic pH, implying that they enter cells by endocytosis. Genome release intermediates of iflaviruses have not been structurally characterized. Here, we show that conformational changes and expansion of iflavirus RNA genomes, which are induced by acidic pH, trigger the opening of iflavirus particles. Capsids of slow bee paralysis virus and sacbrood virus crack into pieces. In contrast, capsids of deformed wing virus are more flexible and open like flowers to release their genomes. The large openings in iflavirus particles enable the fast exit of genomes from capsids, which decreases the probability of genome degradation by the RNases present in endosomes.

## Teaser

To enable genome release, capsids of iflaviruses crack into pieces or open like flowers.

## INTRODUCTION

The family Iflaviridae of insect viruses includes important pathogens of the western honeybee (*Apis mellifera*): deformed wing virus (DWV), sacbrood virus (SBV), and slow bee paralysis virus (SBPV) (*1*). The ectoparasitic mite *Varroa destructor* serves as a vector for honeybee viruses and accelerates their spread within and between colonies (*2*–*5*). DWV causes collapses of bee colonies and is a major threat to the worldwide population of honeybees, endangering the production of one-third of the human diet and the abundance and diversity of wild flowering plants (*6*–*12*).

Viruses from the family Iflaviridae have nonenveloped virions with diameters of 30 to 40 nm (*13*–*16*). The icosahedral capsids of iflaviruses protect single-stranded RNA genomes that are about 10,000 nucleotides long and are polyadenylated at the 3′ end (*17*). The iflavirus genome encodes a single polyprotein, which is co- and posttranslationally cleaved into functional subunits. Capsid proteins VP1, VP2, and VP0, originating from one polyprotein, form a protomer, 60 of which assemble into a pseudo-T = 3 icosahedral capsid. After virion assembly, VP0 subunits are cleaved into VP3 and VP4. VP4 subunits of iflaviruses are short peptides containing 20 to 40 residues (*17*–*19*). Unlike in the related picornaviruses and dicistroviruses, VP4 peptides were not detected in virions of iflaviruses (*14*). Subunits VP3 of DWV and SBPV have 160-residue-long C-terminal extensions, which fold into globular domains protruding from the virion surface (*13*, *16*). The protruding domains contain a cluster of eight conserved residues that constitute a putative hydrolase catalytic site and were speculated to function in virus entry into a cell (*13*, *15*, *16*). VP3 subunits of SBV lack the protruding domains. Instead, SBV virions contain minor capsid proteins attached to the outer capsid surface (*14*). The minor capsid protein of SBV disrupts membranes and may enable the delivery of the virus genome into the cell cytoplasm (*14*).

Capsids protect iflavirus genomes in the extracellular environment, but the virus particles have to release their genomes at the appropriate moment during entry into a host cell. It is assumed that the genome release of iflaviruses is induced by binding to receptors or by exposure to acidic pH in endosomes, as is the case in the better-studied picornaviruses and dicistroviruses (*20*–*26*). These speculations were corroborated by the observation that acidic pH induces the genome release of SBV and SBPV (*14*, *15*). Empty particles of SBPV and SBV, resulting from the acidic treatment, are expanded 2% in diameter relative to the native virions (*14*, *15*). We speculated that genomes of SBPV and SBV may be released through pores that form around the threefold symmetry axes of the expanded particles (*14*, *15*). In contrast, Organtini *et al.* (*27*) suggested that movements of the protruding domains of VP3 subunits of DWV enable the release of its genome through a pore along a fivefold symmetry axis of the capsid. However, genome-release intermediates of iflaviruses were not observed directly, and the genome release mechanism remained unclear.

Here, we present the asymmetric cryo–electron microscopy (cryo-EM) structures of the genome-release intermediates of DWV, SBPV, and SBV and compare their unique and common features. We show that acidic pH induces expansion of the RNA genome in the DWV particle, which, in turn, triggers the swelling and flower-like opening of its capsid. In contrast, capsids of SBPV and SBV fragment to release their genomes. Although the types of capsid opening of the viruses differ, both of the variants provide large gateways for the rapid release of genomes from capsids.

## RESULTS AND DISCUSSION

### Capsids of SBV and SBPV crack into pieces to enable genome release

**Fig. 1:**
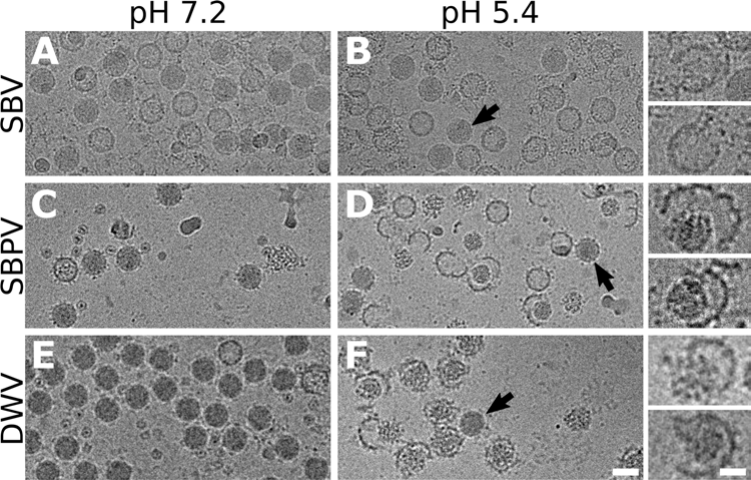
Acidic pH induces capsid opening and genome release of iflaviruses. Cryo–electron micrographs of SBV (**A** and **B**), SBPV (**C** and **D**), and DWV (**E** and **F**). Virions at neutral pH (A, C, and E; the preparations also contain empty particles). Particles exposed to acidic pH (B, D, and F). Arrows indicate particles that remained in native conformation. Insets in (B), (D), and (F) show enlarged images of selected particles. Scale bar, 30 nm (F), 10 nm [inset in (F)].

**Fig. 2:**
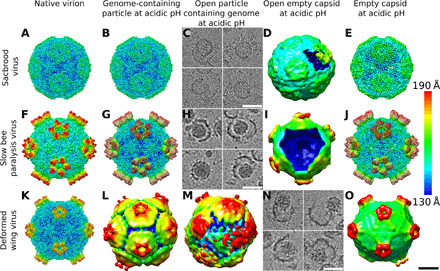
Structural changes in iflavirus particles that enable genome release of SBV, SBPV, and DWV. Native virions (**A**, **F**, and **K**), genome-containing particles at acidic pH (**B**, **G**, and **L**), open particles containing genomes (**C**, **H**, and **M**), open particles without genomes (**D**, **I**, and **N**), and empty capsids resulting from genome release (**E**, **J**, and **O**). Individual panels show cryo-EM reconstructions of particles rainbow colored on the basis of the distance of the particle surface from its center. (C), (H), and (N) show projection images of representative particles, since 3D reconstructions could not be calculated because of structural heterogeneity of the particles. Scale bar, 10 nm.

**Fig. 3:**
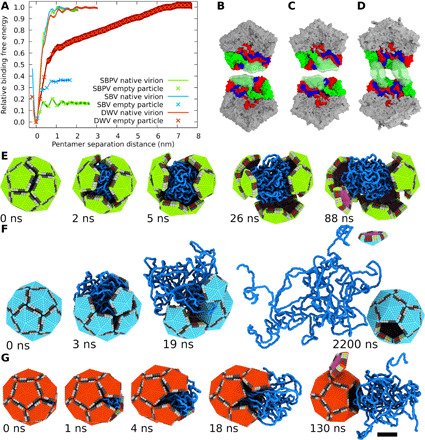
In silico simulation of opening of iflavirus capsids and genome release. (**A**) Binding-free energy of two pentamers of capsid protein protomers across twofold axis in native virions and expanded particles of SBV, SBPV, and DWV. The range of the binding is limited to about 1 nm in the native virions of SBV, SBPV, and DWV and expanded particles of SBV and SBPV. In contrast, the interaction range is 7 nm in the expanded particles of DWV, as indicated by the continuing increase in the relative binding free energy. (**B** to **D**) Interactions of pentamers across twofold axis in acidic pH. The pentamers are viewed from the inside of a particle. Interacting subunits from the two pentamers are shown in the following colors: VP1 in blue, VP3 in green, and VP3 in red. N termini of VP2 subunits of SBV (B) and SBPV (C) do not mediate long-range interactions in contrast to those of DWV (D). Semitransparent green surfaces indicate the limits of movements of the N termini of VP2 subunits. (**E** to **G**) Snapshots from molecular dynamics simulation of genome release. Particles held together by short-range interactions, such as those of SBV (E) and SBPV (F), and long-range interactions corresponding to those of DWV (G). Pentamers of capsid protein protomers are represented as five-sided pyramids and single-stranded RNA as strings of blue beads. Scale bar, 10 nm.

The genome release of iflaviruses can be induced in vitro by exposing the particles to acidic pH (Fig. 1) (*14*, *15*). Protons may enter iflavirus particles, similar to those of picornaviruses, through pores along fivefold symmetry axes of their capsids (*13*–*15*, *28*). Furthermore, capsids of picorna-like viruses are dynamic, and the temporary opening of fissures in the capsid wall may enable an additional exchange of protons (*29*–*31*). The capsids of SBV and SBPV do not expand at acidic pH and retain their native conformation before the genome release (Figs. 1, B and D, and 2, A, B, F, and G; fig. S1; and table S1). Asymmetric cryo-EM reconstruction combined with three-dimensional (3D) classification identified 3% of SBV particles and 2% of those of SBPV that lacked one or a few pentamers of capsid proteins from their capsids (Fig. 2, C, D, H, and I). The remaining 97 and 98% of particles had complete capsids. Furthermore, the electron micrographs showed numerous capsid fragments (Figs. 1, B and D, and 2, C and H). This provides evidence that, at least in vitro, the genome release of SBPV and SBV is enabled by capsid disruption. In silico simulations show that, at acidic pH, the capsid proteins of SBPV and SBV mediated attractive interactions between pentamers to distances shorter than 1 nm (Fig. 3A). The short range of attractive interpentamer interactions makes the capsids of SBPV and SBV prone to fragmentation (Figs. 1, B and D; 2, C and H; and 3, B and C). Similar fragmentation was obtained in in silico simulations of capsid models with the properties of SBPV and SBV, in which the genomes were rapidly released through a wide opening of the capsids (Fig. 3, E and F, and fig. S2). This release is in contrast to the previous hypotheses that genomes of iflaviruses are released from particles as single-stranded RNA through pores along threefold or fivefold axes of the icosahedral symmetry of capsids (*14*, *15*, *27*). These speculations were based on a comparison of the icosahedrally symmetrized structures of capsids of native virions and those of empty particles after the genome release (*13*–*15*, *27*). The use of icosahedral averaging in structure determination prevented the identification of unique asymmetric features, which may be essential for the genome release.

### Particles of DWV open like flowers to release their genomes

**Fig. 4:**
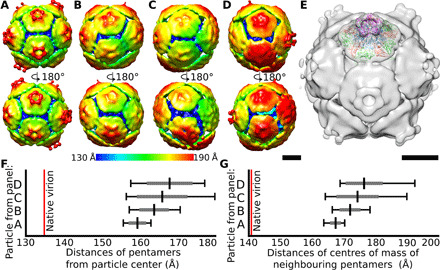
Genome-containing particles of DWV at acidic pH are expanded, asymmetric, and heterogeneous in structure. (**A** to **D**) Asymmetric reconstructions of genome-containing particles of DWV in acidic pH. The particles are rainbow colored on the basis of the distance of the particle surface from its center. Front and rear views of the particles are displayed. Scale bar, 10 nm. (**E**) Fit of PDB model of pentamer of capsid protein protomers from native virion of DWV into asymmetric reconstruction of expanded particle of DWV at acidic pH. Scale bar, 10 nm. (**F**) Plot of distribution of distances of centers of masses of individual pentamers from particle center for reconstructions displayed in (A) to (D). Average values are indicated by black vertical lines, SDs by gray lines, and extreme values by black lines. (**G**) Plot of distances of centers of mass of neighboring pentamers of reconstructions displayed in (A) to (D).

Native virions of DWV at neutral pH are uniform in size with a radius (the distance of the center of the mass of a pentamer from the center of the capsid) of 135 Å (Fig. 1, E and K). In contrast, genome-containing particles of DWV exposed to acidic pH are variable in size, with radii in the range of 155 to 180 Å (Figs. 1F, 2L, and 4; and figs. S3 and S4). The capsids of DWV particles at acidic pH lose their icosahedral symmetry and become structurally heterogeneous (Figs. 1F and 4, A to D, and fig. S3). The shapes of the expanded particles correspond to rotation ellipsoids with a length ratio between the shortest and the longest principal semi-axes of up to 1:1.15 (Fig. 4F). Pentamers of capsid protein protomers maintain a similar structure to that of the native virion (Fig. 4E), but the distances between the centers of masses of adjacent pentamers increase from 141 Å in the native virions to 164 to 192 Å in the expanded particles (Fig. 4G). The pentamers within the expanded capsids are linked by cryo-EM densities positioned either at or next to the icosahedral twofold symmetry axes (Fig. 4, A to E). The limited resolution of the asymmetric reconstructions of expanded particles of DWV prevented the direct identification of the residues of capsid proteins that form the interpentamer links (Fig. 4, A to D). In silico simulations of the expansion of DWV capsid have shown that residues 1 to 61 from the N termini of VP2 subunits can change their conformations and mediate attractive interpentamer interactions up to a distance of 7 nm (Fig. 3, A to D). Therefore, the N termini of VP2 subunits enable the flexible expansion of DWV capsids and the deviation of its overall structure from icosahedral symmetry (Figs. 1F and 4, A to D). Capsid expansion similar in magnitude to that of DWV was observed for equine rhinitis A virus at acidic pH (*32*). However, unlike in DWV, the enlargement of particles of equine rhinitis A virus was accomplished by the rotation of pentamers of capsid protein protomers by 20.9° clockwise about their fivefold axes, indicating a different mechanism of expansion to that of DWV (*32*).

After 30 min of incubation in phosphate-buffered saline (PBS) with pH 5.5 at 34°C, the capsids of 80% of DWV particles were open in a manner reminiscent of the opening of petals of a flower (Figs. 1F, 2, M and N, and 3G). Genomes in the form of nucleocores diffused from the large openings in some of the capsids (Figs. 1F and 2N). Three-dimensional classification determined that 17% of DWV particles lacked one or a few pentamers of capsid protein protomers (Fig. 2M). The opening or removal of a single pentamer from the expanded DWV capsid results in the formation of a pore with a diameter of 190 Å, which is sufficient for the release of the RNA genome without major unwinding of its putative secondary and tertiary structure (Fig. 3G).

### Opening of iflavirus capsids in acidic pH is probably induced by genome expansion

The genome release of iflaviruses, as well as that of picornaviruses and dicistroviruses, is preceded by the formation of activated particles, which have reduced interpentamer contacts relative to the native virions (*22*–*26*). Furthermore, the activation of particles is associated with the structural reorganization of their genomes that change from a uniform distribution in native virions to regions with high and low densities in activated particles (Fig. 1) (*15*, *24*, *33*–*36*). The changes in the organization of the RNA genomes of DWV in acidic pH are connected to the radial expansion of the particles by 15 to 33% relative to the native virions at neutral pH (Figs. 1F; 2, L and M; and 3, A to D and F). In contrast, empty capsids of DWV at acidic pH were only expanded by 5% (Fig. 2O). The capsids of genome-containing particles being larger than those of empty particles provide evidence that at acidic pH, the genome exerts pressure on the inside of the capsid. In contrast, there is no evidence that the genomes of iflaviruses or other picorna-like viruses are packaged under pressure in native virions at neutral pH. Thermal motions cause a fluctuation of the force with which the genome pushes on the inner faces of individual pentamers of capsid protein protomers at acidic pH. We speculate that the force exerted by the genome occasionally exceeds that which holds the capsid together, leading to the opening of the particle and genome release (Fig. 3, E to G). Since changes in genome structure after exposure to acidic pH were also observed in dicistroviruses and picornaviruses (*15*, *24*, *33*–*36*), it is possible that the opening of particles of these viruses is also induced by genome pressure. Single-stranded RNA genomes of picornaviruses were shown to be associated with positively charged polyamines, which enable genome packaging by neutralizing the negative charge of RNA (*37*–*39*). However, the presence of polyamines in particles of iflaviruses has not been experimentally demonstrated. Nevertheless, we speculate that exposure to acidic pH may induce the release of polyamines from particles, resulting in a loss of positive charges that shield the negative charge of the genome. The increased negative charge in the virus particle may lead to the observed changes in genome distribution (Fig. 1, B, D, and F) (*15*, *24*, *33*–*36*).

### Heterogeneity in reactions of iflavirus particles to acidic pH

Three types of behavior of iflavirus particles were observed after exposure to acidic pH: (i) cracked, open, or empty particles that released their genomes, (ii) particles containing genomes with an altered structure of high- and low-density regions, and (iii) particles whose genomes maintained a uniform distribution, just like the native virions at neutral pH (Fig. 1, B, D, and F, and fig. S5). The distinct behaviors of the individual particles under the same conditions may be due to different structures of the RNA genomes packaged inside the virions or due to variations in polyamine content. It is possible that the observed variability in the stability of particles predisposes them for different roles in virus dissemination. The virions that readily release their genomes may efficiently mediate infections of cells within one organism. In contrast, the more stable particles may be better suited to withstand the conditions outside of a host body and transmit the infection between organisms.

### Genome release mechanism

Previous studies of icosahedrally symmetrized particles of iflaviruses, as well as picornaviruses and dicistroviruses, indicated that their genomes were released through pores along twofold, threefold, or fivefold symmetry axes of their capsids (*24*, *40*–*43*). Asymmetric cryo–electron tomography analyses were used to show that the genome exits the poliovirus particle as a single-stranded RNA through a pore close to an icosahedral twofold axis of the capsid (*40*). The genome release of poliovirus was induced by heating the particles to 56°C in a solution with a 65% lower ionic strength than that of the cytoplasm or extracellular liquid in multicellular organisms (*40*). It is possible that the nonphysiological conditions resulted in a disruption of the putative secondary and tertiary structure of the RNA genome of poliovirus and enabled its release through a small pore along the twofold axis of the capsid. Furthermore, the threading of a 10,000-nucleotide-long single-stranded RNA molecule through a narrow aperture in a capsid would be a slow process, which may expose the genomes to degradation by ribonucleases (RNases) in endosomes (*41*, *44*).

**Fig. 5:**
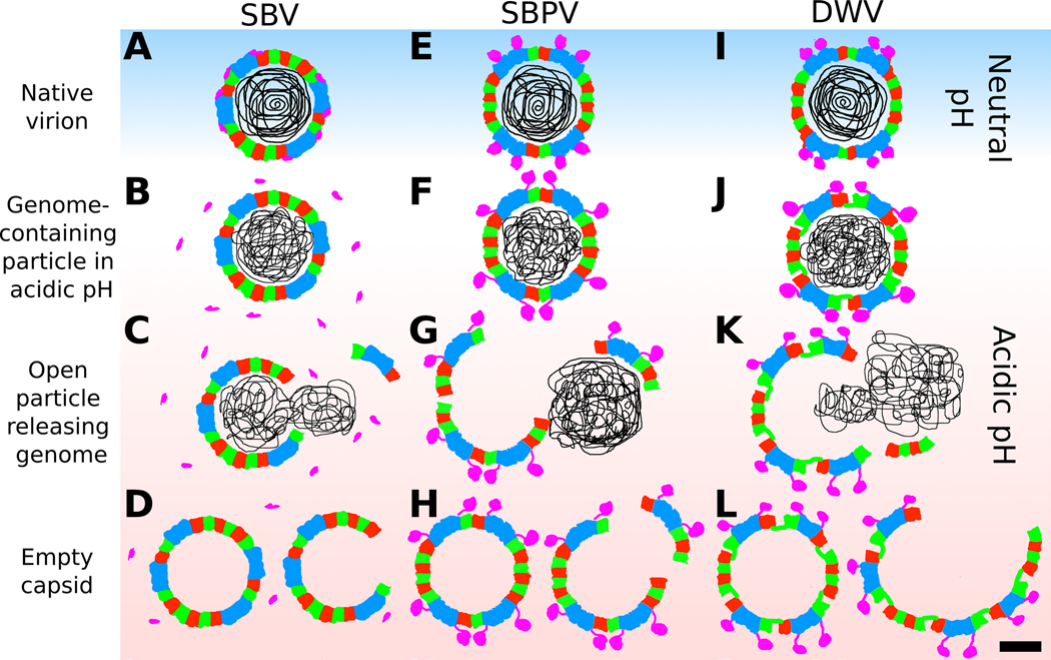
Genome release mechanisms of SBV, SBPV, and DWV. (**A**, **E**, and **I**) Native virions at neutral pH. (**B**, **F**, and **J**) Exposure of the viruses to acidic pH triggers the reorganization of viral genomes and induces detachment of minor capsid proteins from SBV capsid and movements of protruding domains of SBPV and DWV. (J) The particles of DWV expand at acidic pH. Particles of SBV and SBPV crack to release their genomes (**C** and **G**), whereas those of DWV are more flexible and open “like flowers” (**K**). (**D**, **H**, and **L**) The genome release results in open and empty particles. Scale bar, 10 nm.

Structural characterization of particles of SBV, SBPV, and DWV exposed to acidic pH enabled us to propose the mechanism of iflavirus genome release (Fig. 5). Acidic pH induces changes in the structure of virus RNA genomes and probably causes an increase in the pressure inside the capsid (Fig. 5, B, F, and J). Flexible particles of DWV expand, whereas those of SBV and SBPV do not (Fig. 5, B, F, and J). Fluctuations in the force with which the genome pushes on individual pentamers of capsid protein protomers lead to the opening of a particle. Capsids of SBPV and SBV crack into pieces (Fig. 5, C and G), in contrast, those of DWV open like flowers (Fig. 5K). The opening of iflavirus particles enables the rapid exit of genomes from capsids, reducing the possibility of their degradation by RNases (*35*). The genome release of iflaviruses in vivo may be influenced by the interactions of virus particles with receptors. The binding of receptors to virus particles is likely to be asymmetric and may therefore influence the opening of the capsid and thus determine the direction of genome release from the particle. However, the function of the receptors may be limited to enabling the entry of iflaviruses into endosomes. The receptors of iflaviruses are currently unknown, and the determination of their putative function in genome release will require further experimentation.

To infect a cell, iflaviruses have to ensure delivery of their genomes into the cytoplasm, which involves crossing the cytoplasmic or endosome membrane. The infection process of iflaviruses has not been characterized, and we can only speculate about it based on knowledge of better-studied picornaviruses (*21*, *45*). It has been shown that enteroviruses use two alternative mechanisms for genome delivery. There is evidence that human rhinovirus 2 and poliovirus induce the formation of pores in endosome membranes (*21*, *46*–*49*), whereas HRV14 triggers the disintegration of entire endosomes (*50*). Threading the RNA genome through a transmembrane pore would be a slow process and requires the unwinding of the putative secondary structures formed by the RNA. Both of these aspects are contrary to the capsid opening and rapid genome release described here for iflaviruses. Therefore, it is more likely that iflaviruses ensure the delivery of their genomes into the cytoplasm by triggering endosome disintegration. This speculation is indirectly supported by the previous observation that minor capsid proteins of SBV disrupt liposomes, and the protruding domains of DWV and SBPV may have a similar function (*13*–*16*). The opening of capsids as the mechanism of genome release was previously demonstrated for enteroviruses from the family Picornaviridae and proposed for dicistroviruses (*33*, *35*, *51*). Therefore, this mechanism of genome release may be shared by viruses from the order Picornavirales.

## MATERIALS AND METHODS

### Virus purification

Honeybee viruses were purified as described previously (*13*, *16*, *52*). Briefly, 50 experimentally infected honeybee pupae were homogenized with a Dounce homogenizer (piston-wall distance of 0.075 mm) in 30 ml of PBS [Dulbecco’s Phosphate-Buffered Saline Modified, D8537, Sigma-Aldrich; 2.7 mM KCl, 136.9 mM NaCl, 1.5 mM KH_2_PO_4_, and 8.1 mM Na_2_HPO_4_ (pH 7.4)] on ice. The extract was centrifuged at 15,000*g* for 30 min at 10°C. The pellet was discarded, and the supernatant was ultracentrifuged at 150,000*g* for 3 hours in a Ti50.2 fixed-angle rotor (Beckman-Coulter) at 10°C. The resulting pellet was resuspended in PBS in a final volume of 5 ml. MgCl_2_ was added to a final concentration of 5 mM, as well as 20 μg/ml of deoxyribonuclease I and 20 μg/ml of RNase. The solution was incubated at room temperature for 30 min and centrifuged for 15 min at 5500*g* at room temperature. The resulting supernatant was separated using a CsCl (0.6 g/ml) gradient in PBS by ultracentrifugation for 16 hours at 30,000 rpm in an SW41 swinging-bucket rotor at 10°C (Beckman-Coulter). Virus bands were collected by the gentle piercing of ultracentrifuge tubes with an 18-gauge needle. The viruses were buffer exchanged to PBS and concentrated using centrifuge filter units with a 100-kDa molecular mass cutoff.

### Induction of genome release by acidic pH

Freshly purified virus samples (SBPV, SBV, and DVW) in PBS, pH 7.4 (Sigma-Aldrich), at a concentration of 8 mg/ml were mixed with an acidification solution [136 mM NaCl and 50 mM KH_2_(PO)_4_, pH 4.4] in a 1:3 ratio and incubated for 30 min. The resulting mixture had pH 5.5. The mixture (4.2 μl at a virus concentration of 2 mg/ml) was applied onto a holey carbon grid (Quantifoil R2/1, mesh 300; Quantifoil Micro Tools) and vitrified by plunging the grid into liquid ethane using an FEI Vitrobot Mark IV. Grids with the vitrified sample were inspected using a TF20 electron microscope operated at 200 kV.

### Cryo-EM data acquisition, image processing, and single-particle reconstructions

Grids with the vitrified samples were transferred to an FEI Titan Krios electron microscope operated at 300 kV. The microscope was aligned for parallel illumination in nanoprobe mode. The sample in the column of the microscope was kept at −196°C. Images were recorded using an FEI Falcon II direct electron detection camera under low-dose conditions (20 e^−^/Å^2^). Data were collected with defocus values in the range of −1 to −3 μm at a nominal magnification of ×75,000, resulting in a pixel size of 1.063 Å/px. Total acquisition time was 1 s, and each image was saved as seven movie frames. The program motioncor2 was used to align frames from each exposure to compensate for drift and beam-induced motion (*53*). Contrast transfer function parameters were determined using the program Gctf (*54*). The particles for analyses were manually picked using the program e2boxer.py from EMAN2 (*55*). The program RELION 2.1 was used to extract particles from the micrographs (box size of 576 × 576 px) and to further process the dataset (*56*). Particles were binned four times using Fourier cropping in the program Xmipp (*57*), and several rounds of 2D classifications were performed to identify homogeneous sets of particles. Previously published reconstructions of SBV, SBPV, and DWV (Electron Microscopy Data Bank codes DWV-4014, SBPV-4063, and SBV-3863) were low-pass filtered to a resolution of 30 Å and used as initial models for 3D reconstructions. Several rounds of asymmetric (C1) 3D classifications were performed to separate the most homogeneous sets of particles. Refinement according to the “gold standard” was performed using the RELION 3D autorefine procedure. The map was masked with a threshold mask, generated using the RELION mask_create routine. To exclude the possibility of overmasking, the masks were visually inspected, and the shapes of FSC curves of phase-randomized half-maps were checked. The resolutions of the reconstructions were determined as the points at which the values of FSC calculated between the two independent half-sets dropped below 0.143.

### Asymmetric reconstruction of expanded particles of DWV at acidic pH

RELION 3.0 was used to extract expanded particles of DWV from micrographs (box size of 576 × 576 pixels) and to further process the dataset. Particles were binned four times using Fourier cropping in the program Xmipp (final box size of 144 × 144 px), and several rounds of 2D classification were performed to exclude damaged particles. Two-dimensional classification removed 9977 of 126,246 particles. Reconstruction of the native DWV (EMD-3575) was low-pass filtered to a resolution of 30 Å and used as the initial model for 3D reconstruction. Several rounds of asymmetric (C1) 3D classifications were performed to obtain homogeneous classes of particles. Refinement according to the gold standard of twice-binned particles (box size of 288 × 288 px) for each class was performed using the RELION 3D autorefine procedure.

### Measurement of radial expansion of particles of DWV

Structures of pentamers of capsid protein protomers (PDB 5MV6, residues 1 to 61 from the N termini of VP2 subunits were removed from the model) were fitted into asymmetric reconstructions of DWV particles at acidic pH using the fit-in-map tool in the program UCSF Chimera. The position of the center of mass of the fitted pentamer was calculated using the define centroid tool. The position of the center of the particle was determined as the average position of the centers of masses of the 12 fitted pentamers. Distances between the center of the particle and centers of masses of individual pentamers, as well as distances between centers of masses of neighboring pentamers, were calculated.

#### Charge calculation—Monte Carlo simulations

We performed Metropolis Monte Carlo simulations using the Faunus framework (*58*). The spherical cell with a radius of 45 nm contained a virus capsid described with an implicit-solvent coarse-grained model, where every residue was treated as a spherical bead (located at the center of mass of the residue) with a radius derived from the amino acid molecular weight and a common density of 0.9 g/ml. The N and C termini of proteins were represented by special beads. The solvent was treated as a dielectric continuum using the Debye-Hückel approximation with a relative permittivity of 78.7 for the interaction of charged residues (*59*, *60*). The capsid was placed in the middle of the simulation sphere with all degrees of motion frozen. Each amino acid was allowed to change its protonation state by titration move, where protons are allowed to move between the bead and solution. The energy associated with the proton exchange is determined by the change in local electrostatic energy ± (pH − pK_0_)*ln10, where pK_0_ is the negative decadic logarithm of the dissociation constant of the isolated amino acid, and pH is that of the system (*61*). The plus and minus signs in the equation are associated with protonation and deprotonation, respectively. Titratable residues with their pK_0_ values are the following: C terminus (2.6), Asp (4.0), Glu (4.4), His (6.3), N terminus (7.5), Tyr (9.6), Lys (10.4), Cys (10.8), and Arg (12.0). The total number of moves in which there were attempts to protonate/deprotonate residues was at least 1000 per residue in all simulations. The temperature of the ensemble was set to 298 K. We performed calculations of capsids of both native virions and particles in acidic pH with structures determined from the cryo-EM. The average charges of amino acids were determined for DWV at pH 7.4 and pH 5.8, SBV at pH 7.4 and pH 5.8, and SBPV at pH 6.5 and pH 5.5, in the presence of implicit monovalent salt solutions at concentrations of 150 mM.

#### Molecular dynamics simulations

A computationally efficient, coarse-grained MARTINI 2.2 force field was used for calculating the binding free energy between two pentamers of capsid protein protomers in a capsid (*62*–*64*). The structure from all-atom equilibration was converted into a MARTINI model using the martinize.py script. As a consequence of coarse graining, the MARTINI model does not explicitly describe backbone hydrogen bonds. Thus, the secondary structure has to be imposed on the proteins and maintained throughout the simulation. The secondary structure elements were assigned on the basis of the cryo-EM structures using the program DSSP (*65*). To help preserve the higher-order structure, an elastic network was added to the standard martini topology. Harmonic bonds were generated between backbone beads by the martinize.py script using the option −ff elnedyn22. The elastic bonds were not applied to residues exhibiting a high degree of flexibility in the electron density map. Histidines with an average charge higher than 0.4 e^−^, as determined from charge calculations that used Monte Carlo simulations, were changed to the protonated form for further simulations. The conformational changes in the virus capsids induced by acidic pH were also taken into account in molecular dynamics simulations, since the initial structures were based on cryo-EM reconstructions determined under the corresponding conditions. The simulation time step was set to 20 fs. A velocity-rescaling thermostat with a coupling constant of 1.0 ps was used to maintain the temperature at 310 K (*66*). Protein and solvent beads were coupled to separate heat baths to ensure the correct temperature distribution. The pressure was kept at 1 bar with a Parrinello-Rahman barostat with an isotropic coupling scheme with a coupling constant of 12 ps (*67*). All nonbonded interactions were cut off at 1.1 nm, and the van der Waals potential was shifted to zero. The relative dielectric constant was set to 15. Periodic boundary conditions were used. The system consisted of four protomers of capsid proteins in water, NaCl ions at a concentration of 150 mM, and extra ions to neutralize the system. The system size was approximately 19 × 19 × 35 nm.

The umbrella sampling method was used to determine the free energy of binding between two pentamers of capsid protein protomers, as described before (*35*). The reaction coordinate was defined as the *z*-distance between the centers of mass of the two pairs of protomers. We restrained the positions of protomers at the interpentamer interface by the use of harmonic potentials on backbone beads, excluding the flexible residues (table S2) (*35*). Cylindrical flat-bottom positional restraints were applied to the backbone beads of protomers 3 to 4, excluding the flexible residues to keep the protomers from tilting and moving in the *XY* plane. The cylinders were parallel to the *z* axis. The force constant was set to 1000 kJ mol^−1^ nm^−2^, and the radius of all cylinders was 0.3 nm. The reference configuration for the cylindrical flat-bottom position restraint was selected from a 1000-ns equilibration run. For the last 500 ns of the equilibration run, the structures of protomers were averaged. The reference configuration was selected from the trajectory based on the lowest root mean square deviation toward the averaged structure. To analyze the probability distributions of states from each window, we used iterative WHAM implemented in the GROMACS tool gmx wham (*68*, *69*).

#### Phenomenological model

Previously, we developed a phenomenological coarse-grained model of capsids of viruses from the family Picornaviridae (*35*). The capsid is approximated by a regular dodecahedron composed of 12 pentagonal subunits. Each subunit assumes the role of a stable pentamer of capsid protein protomers. The pentamers were assembled from beads organized in three layers. Each pentamer was composed of 317 beads connected by 1311 harmonic bonds with the spring constant of the harmonic potential being 250 kJ/mol. Beads within the capsids only interacted via the harmonic bonds. The capsid included six types of beads, all of which were interacting with the Weeks-Chandler-Anderson repulsive potential with epsilon set to 1.0 (*70*). In addition, beads at the edges of pentamers formed attractive interactions in the range of 0.3 to 5.0 nm based on the properties of SBV, SBPV, and DWV capsids determined using free energy calculations using the MARTINI model. The potentials between pentamers, which were determined by molecular dynamics simulations, were used in the parametrization of our phenomenological model. Therefore, the phenomenological model reflects the capsid changes caused by acidic pH. The attraction only acted between the bead types, which are in contact in the assembled capsid. The interaction decreased to zero with a cos^2^ dependence. There was no attraction between the inner and outer layers. The attraction strength of the middle layer was varied from 5 to 30 kJ/mol, to simulate the genome release of SBV and SBPV. For DWV, two cos^2^ potentials were applied for the middle layer, one with an interaction distance of 1 nm and the other with an interaction distance of 3 or 5 nm. The second interaction distance was selected on the basis of the disruption of binding of one of the two flexible tethers (59 residues from the N terminus of VP2) between two pentamers. At an interpentamer distance of 5 nm, both N termini from one pentamer stopped interacting with the other pentamer. Therefore, we selected these two distances for the interaction range.

To investigate RNA genome release from the capsid, we modeled the RNA as a chain of beads. The beads that were not directly linked in a chain repelled each other. A single bead representing two nucleotides had a radius of 0.6 nm. The beads were connected by a 1.1-nm-long harmonic bond. All the beads were interacting with a shifted truncated Lennard-Jones potential, i.e., Weeks-Chandler-Anderson potential with epsilon set to 1.0 (*70*). Inspired by cryo-EM snapshots, where the genome is compact even after genome release from DWV, we added an attractive potential to the RNA genome in the form of a cos^2^ interaction at each bead with the interaction range of 0.5 nm. To examine the genome release event under various genome cohesion conditions, a range of interaction strengths (0.0, 0.18, 0.3, and 0.42 kcal/mol) was tested (fig. S2).

Simulations were performed in LAMMPS (*71*), with the use of a Langevin thermostat (*72*–*74*). Motion of the center of mass of the entire system caused by the thermostat was eliminated using the option “zero yes.” In addition, the “gjf yes” option was turned on, applying Gronbech-Jensen/Farago time discretization for the Langevin model to enable longer time steps while still producing the correct Boltzmann distribution of atom positions (*74*). The viscous damping term was set to 10,000 time steps. The reduced temperature in our simulations was T* = 1 *k*_B_*T*. The box size of 150 × 150 × 150 nm was constant and the same for all simulations. The simulation protocol was as follows: First, the capsid was generated from pentamers, and then the chain representing the genome was generated within the capsid using a random walk. The equilibration started with the chain equilibration alone, which was simulated with Langevin dynamics for 10^8^ steps, while the capsid shell was motionless. The second step was the equilibration of both the capsid and the genome. The attraction between a pentamer of capsid proteins and genome began at 35 kJ mol^−1^ and was gradually decreased by a rate of 0.25 kJ mol^−1^ every 200,000 time steps to generate a set of starting configurations. The genome release simulations were repeated nine times for each set of parameters (attraction strength and attraction range). The simulations were terminated after 10^9^ time steps or sooner if the genome release occurred.

## Supplementary Material

http://advances.sciencemag.org/cgi/content/full/7/1/eabd7130/DC1

Adobe PDF - abd7130_SM.pdf

Capsid opening enables genome release of iflaviruses

## SUPPLEMENTARY MATERIALS

Supplementary material for this article is available at http://advances.sciencemag.org/cgi/content/full/7/1/eabd7130/DC1

## Appendix

View/request a protocol for this paper from *Bio-protocol*.
