# Supplementary material for: Capsid opening enables genome release of iflaviruses
Source: Sci Adv. 2021 Jan 1;7(1):eabd7130. doi: 10.1126/sciadv.abd7130 (PMC7775750; doi:10.1126/sciadv.abd7130)
Supplement: http://advances.sciencemag.org/cgi/content/full/7/1/eabd7130/DC1 [file supp_7_1_eabd7130__index.html]

Science Advances | Science AdvancesAAASSearchScience AdvancesMenu

## Supplementary Materials

# Capsid opening enables genome release of iflaviruses

Karel Škubník, Lukáš Sukeník, David Buchta, Tibor Füzik, Michaela Procházková, Jana Moravcová, Lenka Šmerdová, Antonín Přidal, Robert Vácha, Pavel Plevka

Download Supplement

**This PDF file includes:**

- Figs. S1 to S5
- Tables S1 and S2

**Files in this Data Supplement:**

- Adobe PDF - abd7130\_SM.pdf
