# Supplementary material for: Capsid opening enables genome release of iflaviruses
Source: Sci Adv. 2021 Jan 1;7(1):eabd7130. doi: 10.1126/sciadv.abd7130 (PMC7775750; doi:10.1126/sciadv.abd7130)
Supplement: http://advances.sciencemag.org/cgi/content/full/7/1/eabd7130/DC1 [file supp_7_1_eabd7130__1.pdf]

Science Advances
AAAS

advances.sciencemag.org/cgi/content/full/7/1/eabd7130/DC1

Supplementary Materials for

Capsid opening enables genome release of iflaviruses

Karel Škubník, Lukáš Sukeník, David Buchta, Tibor Füzik, Michaela Procházková, Jana Moravcová, Lenka Šmerdová, Antonín Přidal, Robert Vácha, Pavel Plevka*

*Corresponding author. Email: pavel.plevka@ceitec.muni.cz

Published 1 January 2021, *Sci. Adv.* **7**, eabd7130 (2020)
DOI: 10.1126/sciadv.abd7130

This PDF file includes:

Figs. S1 to S5
Tables S1 and S2

Supplementary figures:

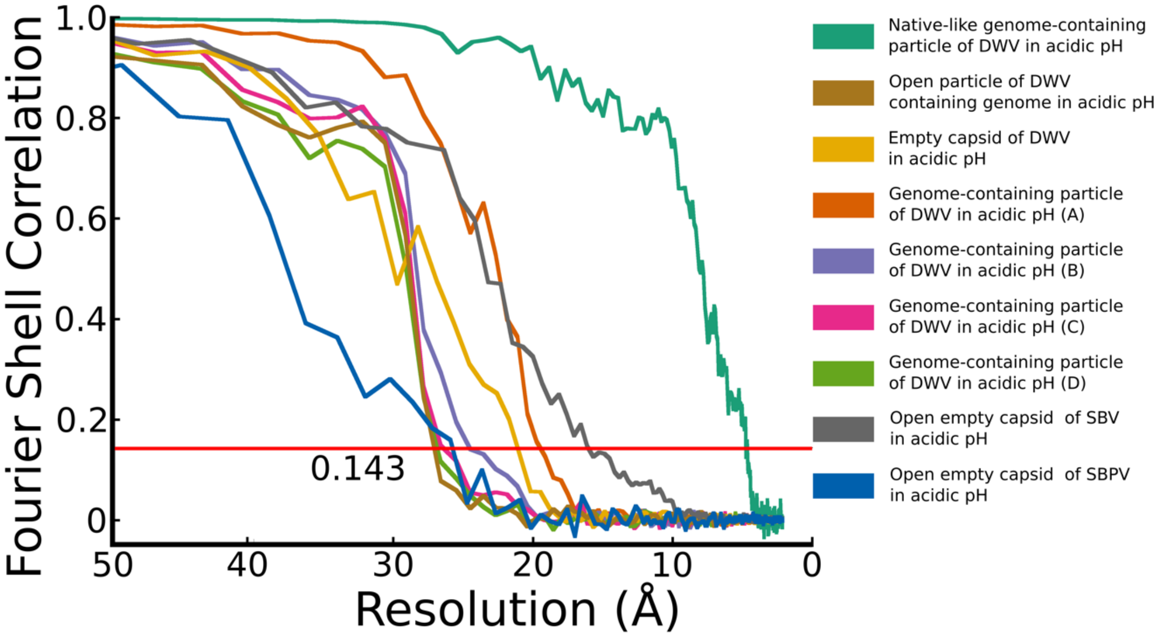

Fig. S1. FSC curves of cryo-EM reconstructions.

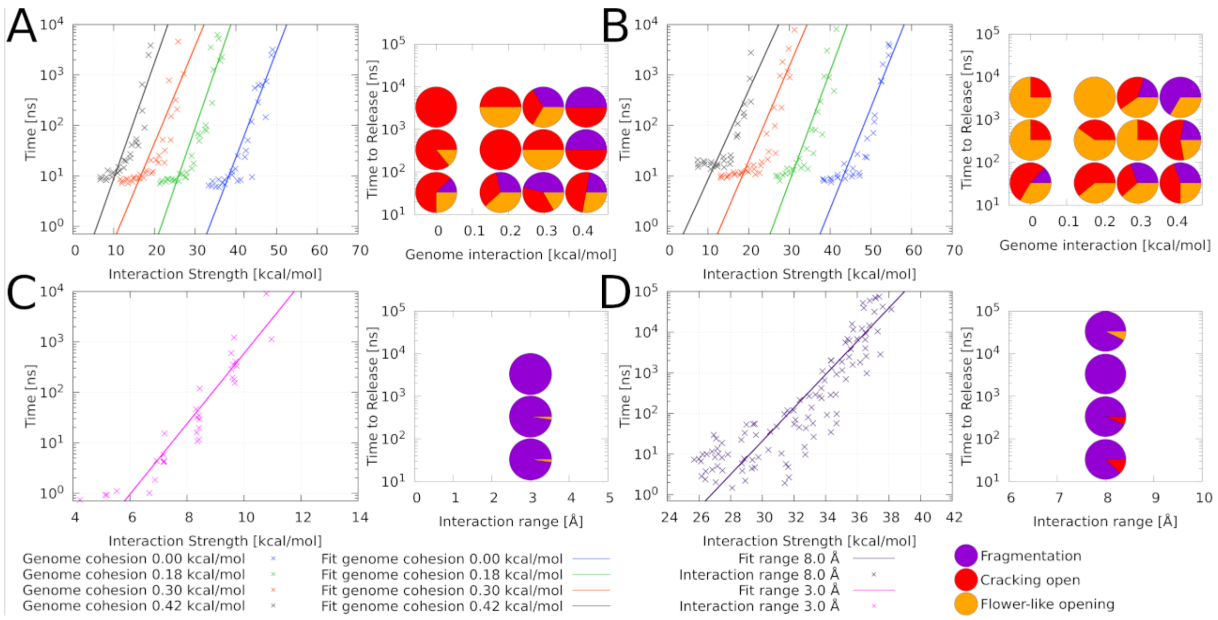

Fig. S2. Stability and types of observed genome release pathways of virions. Models representing DWV particles with the interaction ranges of pentamers of 3 nm (A) and 5 nm (B). Various cohesive attractions (0.0, 0.18, 0.30, 0.42 kcal/mol) of genomes in virions of DWV were investigated. (C) Model representing SBPV particle with an interaction range of 0.3 nm and genome cohesivity 0.42 kcal/mol. (D) Model representing SBV particle with an interaction range of 0.8 nm and genome cohesivity 0.42 kcal/mol. On the left side, is the graph of capsid stability with time to genome release as a function of pentamer-pentamer interaction strength obtained from simulations and fitted with Arrhenius equation. The distributions of release pathways are shown on the right. Fragmentation release is defined as the breaking of the capsid into more than two fragments during the genome release. Capsid cracking is defined by the splitting of the capsid in the middle. The flower-like opening is defined as a flexible opening of pentamers from one hemisphere whereas the other hemisphere remains stable. For both SBV and SBPV, the majority of release paths is fragmentation, while the release from DWV is dominated by the flower-like opening pathway for genome cohesion below 0.4 kcal/mol. The less realistic DWV model with shorter interaction of 3 nm favors the cracking of capsids.

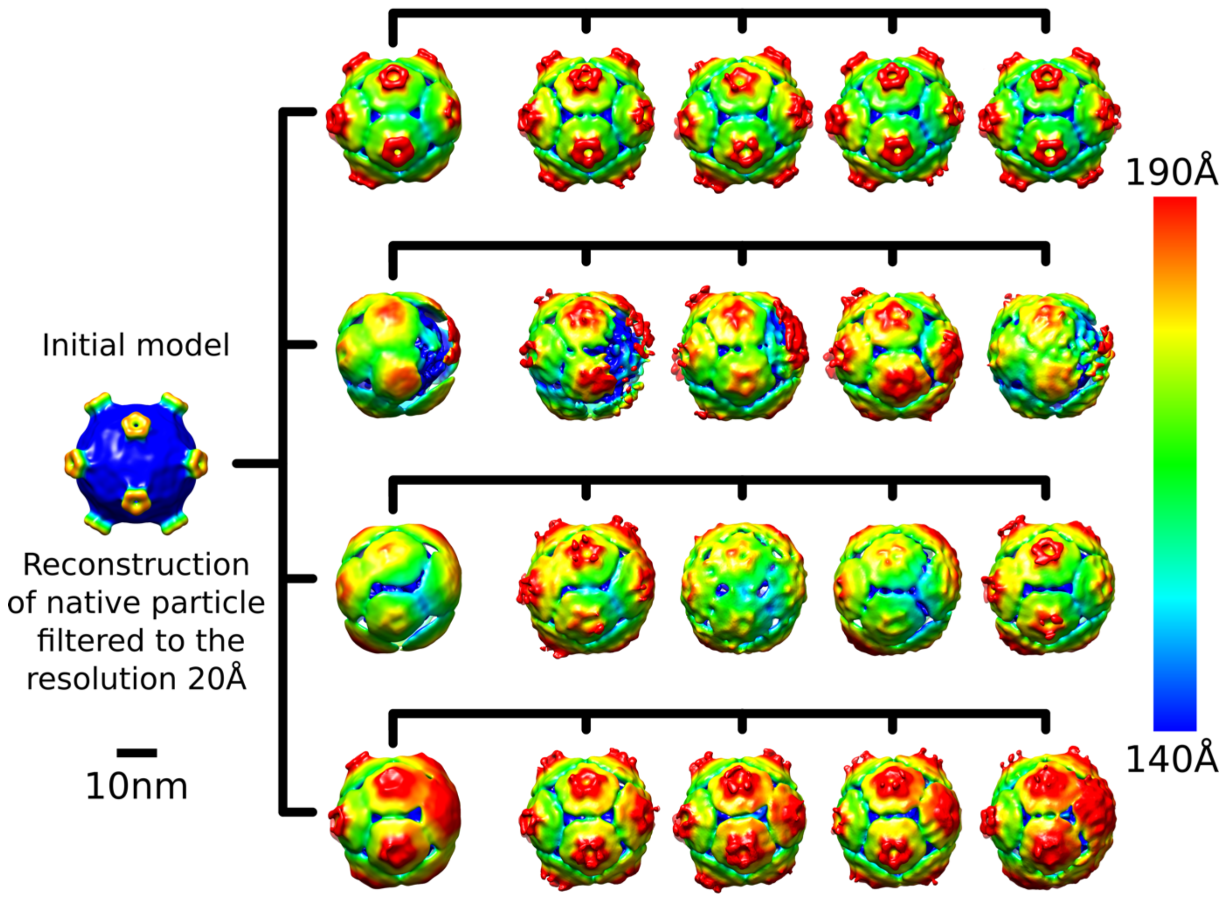

Fig. S3. Three-dimensional classification of expanded genome-containing particles of DWV. The classification was initiated using the reconstruction of the native virion of DWV low-pass-filtered to a resolution of 20 Å. The classification was performed in two rounds: initially four classes were obtained, then each of the classes was classified into four sub-classes. The particles are rainbow-colored based on the distance of the particle surface from its center. Scale bar indicates 10 nm.

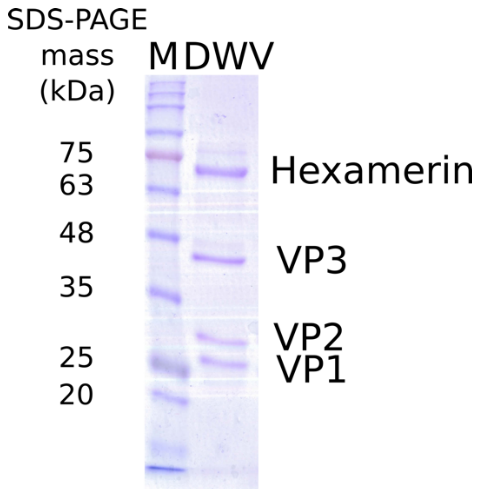

Fig. S4. SDS gel of DWV purified on CsCl density gradient and used for cryo-EM sample preparation. The preparation of the virus was contaminated with the honeybee protein hexamerin with a molecular weight of 68 kDa. The identity of the proteins in the sample was verified using mass spectrometry. The gel was stained using Coomassie Brilliant Blue R-250 Dye. M indicates the line with the marker: Pre-stained Protein Ladder – Broad molecular weight (10-245 kDa) (ab116028). The straight horizontal lines are due to the scanning of the gel using the Xerox WorkCentre 6605 machine.

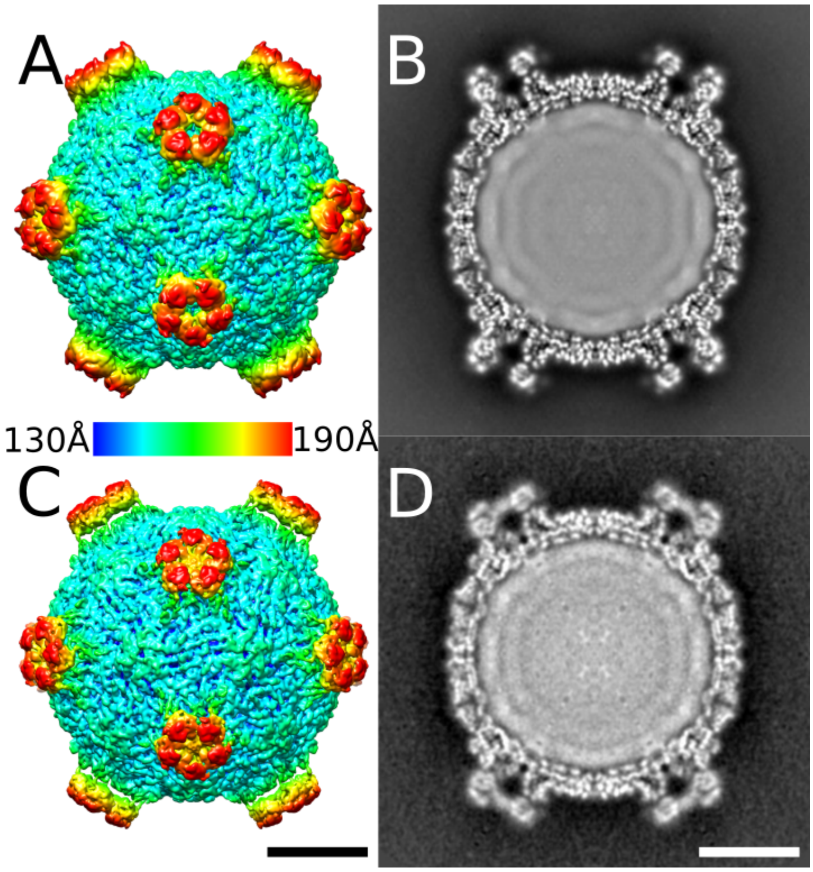

Fig. S5. Structure of native virion of DWV at neutral pH and genome-containing particle of DWV (A,B), which retained native-like conformation after exposure to acidic pH (C,D). (A,C) Surface renderings of cryo-EM reconstructions. The densities are rainbow-colored based on the distance of the surface from the particle center. (B,D) Central sections of cryo-EM densities. White color indicates high density. Scale bars indicate 10 nm.

Supplementary table 1. Cryo-EM structure quality indicators.

	Native-like genome-containing particle of DWV at acidic pH	Open particle of DWV containing genome at acidic pH	Empty capsid of DWV at acidic pH	Genome-containing particle of DWV at acidic pH (A)	Genome-containing particle of DWV at acidic pH (B)	Genome-containing particle of DWV at acidic pH (C)	Genome-containing particle of DWV at acidic pH (D)	Open empty capsid of SBV at acidic pH	Open empty capsid of SBPV at acidic pH
Magnification	75 000	75 000	75 000	75 000	75 000	75 000	75 000	75 000	75 000
Voltage [kV]	300	300	300	300	300	300	300	300	300
Pixel size [Å]	1.063	2.126	1.063	2.126	2.126	2.126	2.126	1.063	1.063
Symmetry	I4	C1	C1	C1	C1	C1	C1	C1	C3
No. of particles	933	4 758	3 177	23 031	7 011	5 575	3 578	7 549	1 737
Initial model	DWV virion low pass filtered to 30Å	DWV virion low pass filtered to 30Å	DWV virion low pass filtered to 30Å	DWV virion low pass filtered to 30Å	DWV virion low pass filtered to 30Å	DWV virion low pass filtered to 30Å	DWV virion low pass filtered to 30Å	SBV virion low pass filtered to 30Å	SBPV virion low pass filtered to 30Å
Map resolution [Å]	4.8	28	21	20	25	27	27	16	27
FSC threshold	0.143	0.143	0.143	0.143	0.143	0.143	0.143	0.143	0.143
EMDB/PBD	11815/7AL3	11703	11704	11705	11706	11709	11711	11714	11716

Supplementary table 2. List of flexible residues used in in silico simulations of the studied capsids.

	DWV native	DWV activated
Chain A	1, 252-258	1-22, 251-258
Chain B	1, 250-253	1-59, 96-102, 249-253
Chain C	95-97, 399-416	1-6, 395-416
	SBV native	SBV activated
Chain A	243-247	1-14, 243-247
Chain B	1-4, 243	1-43, 243
Chain C	274-280	273-280
Chain D	1-21, 48	1-21, 48
	SBPV native	SBPV activated
Chain A	1-5, 186-187, 247-266	1-12, 182-191, 246-266
Chain B	92-100	1-35, 191-200, 261
Chain C	330, 394, 414-430	1, 73-75, 218-221, 264-430
